# Transverse propagation in an expanded PSpice model for cardiac muscle with gap-junction ion channels

**DOI:** 10.1186/1475-925X-5-46

**Published:** 2006-07-28

**Authors:** Lakshminarayanan Ramasamy, Nicholas Sperelakis

**Affiliations:** 1 Dept. of Molecular & Cellular Physiology University of Cincinnati College of MedicineCincinnati, OH 45267-0576, USA; 2 Dept. of Electrical Computer Engineering and Computer ScienceUniversity of Cincinnati College of Engineering Cincinnati, OH 45219, USA

## Abstract

Transverse propagation was previously found to occur in a two-dimensional model of cardiac muscle using the PSpice software program for electronic circuit design and analysis. Longitudinal propagation within each chain, and transverse propagation between parallel chains, occurred even when there were no gap-junction (g-j) channels inserted between the simulated myocardial cells either longitudinally or transversely. In those studies, there were pronounced edge (boundary) effects and end-effects even within single chains. Transverse velocity increased with increase in model size. The present study was performed to examine boundary effects on transverse propagation velocity when the length of the chains was held constant at 10 cells and the number of parallel chains was varied from 3 to 5, to 7, to 10, and to 20. The number of g-j channels was either zero, both longitudinally and transversely (0/0), or 100/100. Some experiments were also made at 100/0, 1/1, and 10/10. Transverse velocity and overall velocity (both longitudinal and transverse components) was calculated from the measured total propagation time (TPT), i.e., the elapsed time between when the first action potential (AP) and the last AP crossed the zero potential level. The transverse g-j channels were placed only at the ends of each chain, such that propagation would occur in a zigzag pattern. Electrical stimulation was applied intracellularly between cells A1 and A2. It was found that, with no g-j channels (0/0), overall velocity increased almost linearly when more and more chains were placed in parallel. In contrast, with many g-j channels (100/100), there was a much flatter relationship between overall velocity and number of parallel chains. The difference in velocities with 0/0 channels and 100/100 channels was reduced as the number of chains was increased. In conclusion, edges have important effects on propagation velocity (overall and transverse) in cardiac muscle simulations.

## Background

Successful transmission of excitation from one myocardial cell to the next contiguous myocardial cell can occur without the necessity of gj-channels between the cells. This has been demonstrated to be possible in theoretical and modeling studies by Sperelakis and colleagues [1-4]. In addition, the essential phenomenon in electric field (EF) transmission has been confirmed by other laboratories, [5-7]. As was stated in the 1977 paper of Sperelakis and Mann [1], for the EF mechanism to work successfully, the junctional membrane must be more excitable than the contiguous surface sarcolemma. The fact that the junctional membranes (i.e., the intercalated disks) have a higher concentration (density) of fast Na^+ ^channels than the surface sarcolemma [6,8-10] should cause them to be more excitable than the surface membrane.

Kucera et al. [10] did a simulation study of cardiac muscle in which they determined how conduction velocity varied as a function of the gap-junction resistance (i.e., number of gj-channels) while varying the fraction of fast I_Na _channels located in the junctional membranes. For a 10 nm (100 Å) cleft width and 50% of the I_Na _channel located in the junctional membranes, they found that conduction still occurred at a velocity of about 20 cm/sec when cell coupling was reduced to 10% of normal. Velocity was about 10 cm/sec when coupling was 1% of normal. Consistent with our previous report [11] they observed that the EF mechanism actually slowed velocity by a significant amount when there was strong ("normal") coupling.

In biological studies on connexon43 knockout mice, and therefore virtually absent in gj-channels in their hearts, it was shown that propagation velocity only was slowed, but not blocked [12-15]. And these mice survive. Therefore, it seems clear that the presence of gj-channels is not essential for propagation of excitation in the heart. But when hearts do contain gj-channels (e.g., mammals and adult birds), propagation velocity is speeded up. The PSpice simulation studies suggest that too many gj-channels (e.g., more than 100 channels per junction) causes the propagation velocity to greatly exceed the physiological range. In biological experiments, Rohr et al. [7] found that partial uncoupling of the heart (using 10 μm palmitoleic acid) actually improved impulse conduction by converting unidirectional block to bidirectional propagation (although slower).

Transverse propagation was previously found to occur in a two-dimensional model of cardiac muscle using the PSpice software program for electronic circuit design and analysis [16-19]. Longitudinal propagation within each chain and transverse propagation between parallel chains occurred even when there were no gap-junction (g-j) channels inserted between the simulated myocardial cells either longitudinally or transversely. The transverse propagation is probably mediated by the interstitial potential that develops [16-20]. In previous studies, there were pronounced edge (boundary) effects and end-effects even within single chains [16,20]. Transverse velocity increased with increase in model size. The present study was performed to examine boundary effects on transverse propagation velocity when the length of the chains was held constant at 10 cells and the number of parallel chains was varied from 3 to 20.

## Methods

**Figure 1:**
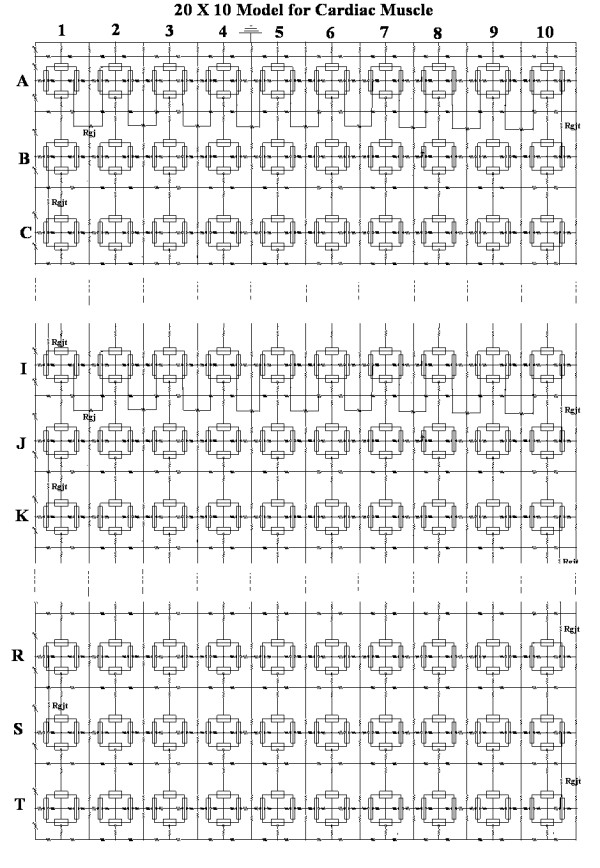
The model for cardiac muscle used for PSpice analysis of propagation. Each chain contained 10 cells, connected longitudinally by cell junctions. The number of chains placed in parallel was varied from 3 (chains A-C), to 5 (chains A-E), to 7 (chains A-G), to 10 (chains A-J), and to 20 (chains A-T). The longitudinal resistance between chains (R_ol2_) had a standard value of 200 KΩ.

The methods used and the modeling with PSpice were given in great detail in previous papers [21,22]. In brief, each myocardial cell was simulated by four basic circuit units; two for the surface sarcolemma (one depicted upwards and one downwards) and one for each junctional membrane at the two ends of the cell (Fig. 1). The cell junctions contained a transverse resistance, the radial resistance of the junctional cleft (R_jc_). The standard value used for R_jc _was 25 MΩ (two 50 MΩ resistors in parallel).

A shunt resistance (R_gj_, resistance of the gap junction channels) was placed across each cell junction, i.e., from one cell interior to the next (Fig. 1). It was assumed that each gj-channel had a conductance of 100 pS, so R_gj _was 100 MΩ when 100 gj-channels were inserted, and 100,000 MΩ when no gj-channels were present.

When present, the transverse gj-channels were placed only at the ends of each chain, i.e., between cells A10 and B10, B1 and C1, C10 and D10, D1 and E1, etc. Thus, propagation could occur in a zigzag pattern [23]. The length of the chains was held constant at 10 cells (cell 1, cell 2, etc), and the number of chains in parallel was varied from 3 to 20 (namely, 3, 5, 7, 10, and 20) (chain A, chain B, etc). Stimulating pulses were applied intracellularly between cells A1 and A2 (rectangular current pulses of 0.25 nA amplitude and 0.25 ms duration).

Overall velocity (θ_ov_) was calculated from the measured TPT, and assuming that the AP impulse traveled down each chain of 10 cells in succession. The myocardial cells were assumed to be cylinders 150 μm long and 16 μm in diameter. For example, in the 10 × 10 model, the following equation would apply:



Then, the transverse velocity (θ_tr_) was calculated from the following equation:



Hence, for given TPT, the overall velocity would be greater by 93.75 × the transverse velocity.

## Results

**Figure 2:**
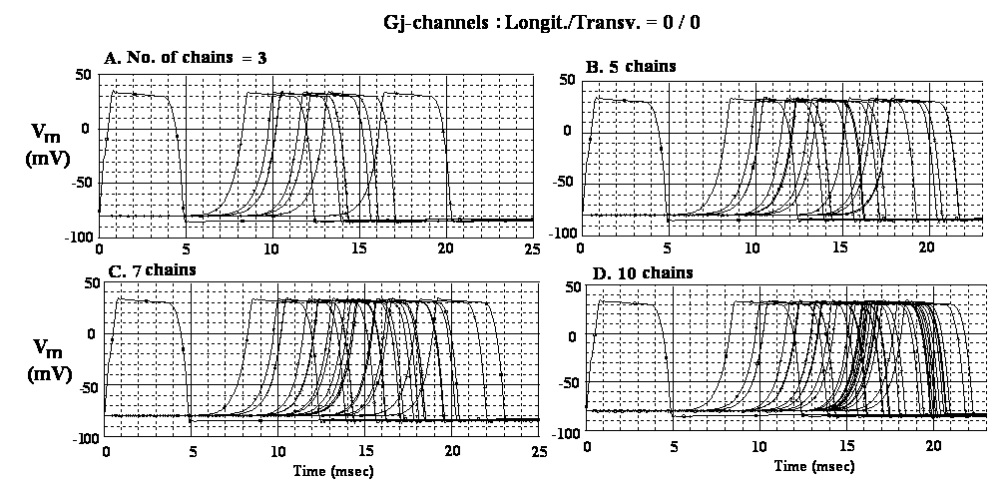
AP records obtained in the model for cardiac muscle when there were no gj-channels, either longitudinally or transversely (0/0). A: 3 chains in parallel. B: 5 chains in parallel. C: 7 chains in parallel. D: 10 chains in parallel. Voltage probes were placed only in cells 1, 5 and 10 of each chain in order to reduce the complexity.

The AP records obtained when there were no gj-channels (0/0) are shown in Figure 2 for 3 parallel chains (A), 5 parallel chains (B), 7 chains (C), and 10 chains (D). Voltage markers were placed in only cells 1, 5, and 10 of each chain to reduce the complexity. The measured TPT values were 15.6 ms (A), 17.1 ms (B), 18.4 ms (C), and 17.6 ms (D). The calculated transverse velocity values were, respectively, 0.21, 0.37, 0.52, 0.82 and 1.69 cm/sec (Table 2). The calculated overall velocity values were, respectively, 27.9 cm/sec, 43.0 cm/sec, 56.3 cm/sec, and 84.4 cm/sec (Table 2).

**Figure 3:**
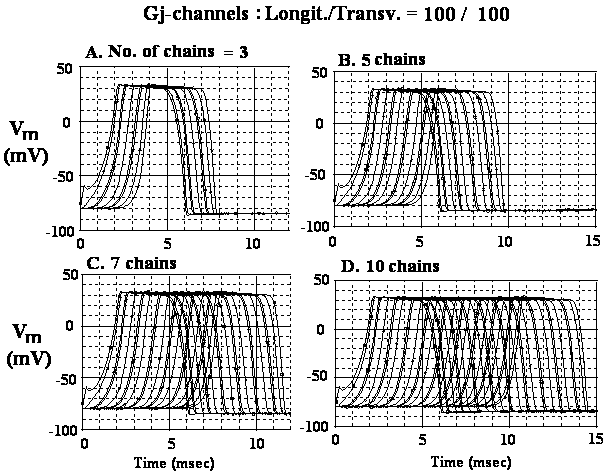
AP records obtained in the cardiac muscle model when there were many (100) gj-channels, both longitudinally and transversely (100/100). The transverse gj-channels were placed only at the ends of the chains (e.g., at cells A10-B10, cells B1-C1, cells C10-D10, etc), giving a zigzag pattern. Voltage probes were placed only in cells 1, 5, and 10 of each chain. A: 3 chains in parallel. B: 5 chains in parallel. C: 7 chains in parallel. D: 10 chains in parallel.

The AP records recorded when there were 100 gj-channels, both longitudinally and transversely (100/100) are shown in Figure 3 for 3 parallel chain (A), 5 parallel chain (B), 7 chains (C), and 10 chains (D). Voltage markers were present in only cells 1, 5, and 10 of each chain. The measured TPT values were 1.9 ms (A), 3.9 ms (B), 5.8 ms (C), and 8.6 ms (D). The calculated transverse velocity values were, respectively, 1.68, 1.64, 1.66, 1.67 and 2.00 cm/sec (Table 2). The calculated overall velocity values were, respectively, 229, 189, 178, and 173 cm/sec (Table 2). Note that the latent period to the first AP was markedly increased compared to that in Figure 2 as expected, because with high cell coupling, it is more difficult to produce excitation with a fixed stimulus.

**Figure 4:**
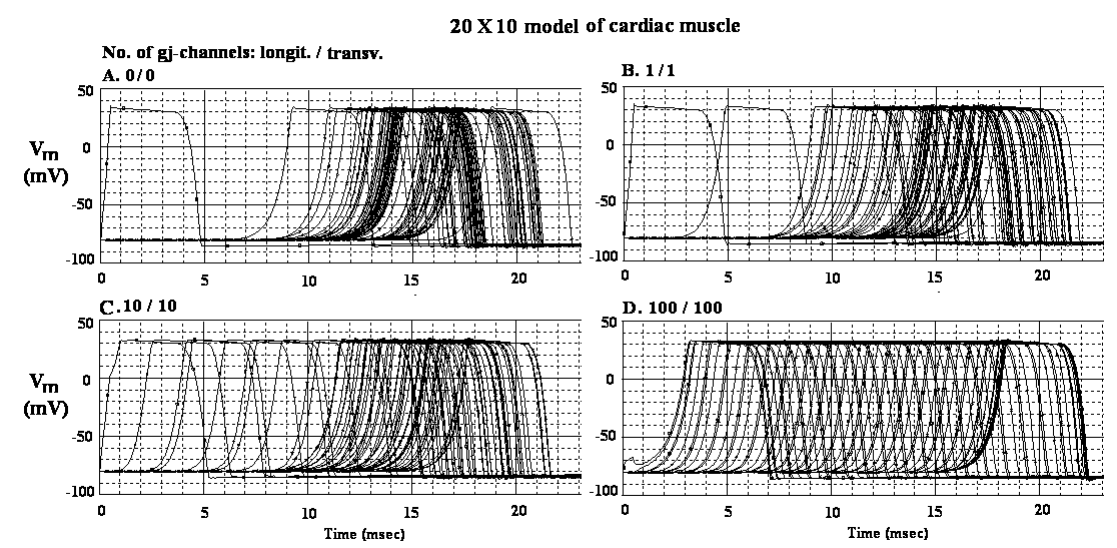
Cardiac action potential records obtained from the 10 × 20 model (10 cells per chain, 20 parallel chains) for different numbers of gj-channels. The number of gj-channels, longitudinal to transverse, is indicated as a ratio. A: 0/0 channels. B: 1/1 channels. C: 10/10 channels. D: 100/100 channels. Voltage probes were placed only in the end cells of each chain (cells 1 and 10), to reduce complexity.

The AP records obtained in the 10 × 20 model are shown in Figure 4 for different degrees of cell coupling: 0/0 (A), 1/1 (B), 10/10 (C), and 100/100 (D). The voltage markers were placed in only the two end cells of each chain (cells 1 and 10), to reduce complexity. The TPT values were 18.0 ms (A), 17.3 ms (B), 17.0 ms (C), and 15.2 ms (D). The calculated transverse velocity values were, respectively, 17, 18, 18, and 18 cm/sec. The calculated overall velocity values were, respectively, 166, 173, 176, and 196 cm/sec. The latent period of the first AP was increased as more and more gj-channels were added, as expected (panels B – D). The latent period in Fig 4D is much larger than that in Fig 2D, for the same number of gj-channels, because the size of the interconnected network has been doubled (10 parallel chains to 20).

**Figure 5:**
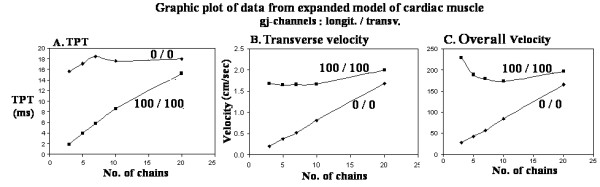
Graphic summary of the results obtained for no gj-channels (0/0) or 100 gj-channels (100/100). The number of parallel chains is given on the abscissa. A: TPT measured. B: Transverse velocity calculated from the TPT and distance traveled. C: Overall velocity calculated from the TPT and distance traveled. The myocardial cells were assumed to be 150 μm in length and 16 μm in diameter.

A graphic summary of the data for 0/0 and 100/100 gj-channels are given in Figure 5. Panel A gives the measured TPT values as a function of the number of parallel chains, panel B gives the calculated transverse velocity as a function of number of parallel chains, and panel C gives the calculated overall velocity as a function of number of parallel chains. Note that both velocities increase almost linearly with increase in number of chains.

## Discussion

We had previously assessed the effect of size of model on transverse propagation velocity by comparing the transverse velocity on square models of different sizes, namely 3 × 3, 5 × 5, and 7 × 7. However, a rectangular model, where one dimension is held constant at 10 cells, should give a more accurate assessment of the boundary/edge effects (e.g., see Wang et al. [20]) on transverse velocity. Therefore, 10 × 3, 10 × 5, 10 × 7, 10 × 10, and 10 × 20 models were used for comparison. In addition, the transverse velocities were compared at two different degrees of cell coupling, namely 0/0 and 100/100. It was found that transverse velocity (Fig. 5B) and overall velocity (Fig. 5C) both increased almost linearly with increase in number of parallel chains when there were no gj-channels (0/0). In contrast, when there was high cell coupling (100/100), the transverse velocity (Fig. 5B) and overall velocity (Fig. 5C) were nearly flat. Consistent with this, the TPT for 100/100 increased almost linearly, and that for 0/0 was almost flat (Fig. 5A).

The conclusion that there is lesser and lesser effect of gj-channels in larger and larger networks is consistent with the fact that the ratios of TPT, 0/0 to 100/100, and of transverse velocity and overall velocity becomes lower and lower as the size of the network is increased (Table 2).

Figure 5 also indicates that the facilitory or potentiating effect of many gj-channels on transverse velocity and overall velocity, as compared to the pure electric field (EF) mechanism (0/0), becomes less and less as the network is increased in size. That is, the two curves are converging. This suggests that the EF mechanism alone can account for the measured propagation velocities in the intact myocardium.

Figure 5, in addition, indicates that the boundary/edge effects are less important when there is high cell coupling, because transverse velocity and overall velocity are relatively flat with increase in number of parallel chains. In contrast, when there are no gj-channels (0/0), transverse velocity and overall velocity keep increasing with increase in number of parallel chains [24]. This suggests that the edge effects act to slow velocity, in agreement with our previous report [16,19]. Presumably, velocity should saturate or level off when the number of parallel chains is increased sufficiently.

Related to this is the fact that the propagation velocity in the first half of the network (chains A-J) is speeded up when another 10 chains are added in parallel (chains K-T), as can be seen in Figure 2. Thus, adding the second half of the network speeds propagation in the first half because the edge effect is pushed further downstream.

Although Figure 5 and Table 2 show velocities that are higher than those found physiologically in the intact heart, the absolute values can be reduced by decreasing the excitability of the basic circuit units. We previously demonstrated that decreasing the excitability of the basic units slows propagation velocity [25].

When there were no gj-channels (0/0), there was usually a large delay between the first AP (from stimulated cell A1 – A2) and the second AP recorded. This delay could be reduced by increasing R_BT_, the bundle termination resistances at the two ends of the bundle. Adding a capacitance in series with R_BT _also acted to reduce this delay. We believe that this peculiar delay is due to an edge effect at the top of the network. When a cell pair near the middle of the network was stimulated (instead of cells A1 – A2), then no such delay was observed.

Transverse propagation is known to occur physiologically in cardiac muscle through the thickness of the ventricular wall (i.e., transmurally), from the endocardial surface to the epicardial surface [26,27]. The transmural conduction velocity in canine heart was substantially faster in the endo to epi direction (48 ± 6 cm/sec) than in the reverse direction (37 ± 6 cm/sec) [26]. There were heterogenties in number of gap junctions through the wall thickness. Since the physiological transverse conduction velocity is much higher than what we have obtained for transverse transmission by the EF mechanism, there must be gap junctions oriented in the transverse direction.

In summary, the present study demonstrates that strong edge effects affect transverse velocity and overall velocity when there are no gj-channels (0/0 category). Thus, propagation by the EF mechanism is slowed by edges. In contrast, when there is strong cell coupling, transverse velocity and overall velocity are not much affected by edge effects. If the network is large enough, propagation by the EF mechanism is almost as fast as in the case of high coupling.

### Study limitations

The present study has some limitations. (1) First, the 20 × 10 model size used is relatively small as compared to regions of the intact heart. Therefore, the importance of the edge effects described here is unclear with respect to relevance to the whole heart. (2) Second, the computational method used here has some limitations in comparison to some other methods that have been used [28-31]. (3) Third, the placement of the transverse gj-channel only at the ends of the chains, to create a zigzag pattern of transverse propagation, may not represent the situation in normal intact heart. However, a zigzag pathway has been observed in canine atria under pathophysiological conditions [23]. (4) Fourth, when there were no longitudinal or transverse gj-channels, the junctional delays were longer than those reported experimentally [6,31]. (5) The importance and relevance of edge effects in intact heart is not clear at the present time, but the ventricular wall contains several layers of fibers running in different directions.

**Table 1:** Parameter values used under standard conditions.

**Parameters**	**Values**
C_m_	300 fF (30)
R_K_	71 MΩ(710)
R_Na_	710 MΩ(7100)
E_K_	-94 mV
E_Na_	+60 mV
R_d_	5000 MΩ
C_d_	30 pF
R_or_	1.0 KΩ
R_ol_	1.0 KΩ
Ri	500 KΩ
R_jc_	25 (50/2) MΩ
R_BT_	200 KΩ

Values for the junctional units are given in parentheses

C_m _= Total cell capacitance

R_K _= Potassium resistance

R_Na _= Sodium resistance

E_K _= Potassium equilibrium potential

E_Na _= Sodium equilibrium potential

R_d _= Resistance in delay circuit for second black-box to bring about AP repolarization

C_d _= Capacitance in delay circuit for second black-box bring about AP repolarization

R_or _= Radial resistance of external fluid

R_ol _= Longitudinal resistance of external fluid

R_i _= Longitudinal resistance of intracellular fluid

R_jc _= Radial resistance of junctional cleft

R_BT _= Bundle termination resistance

**Table 2:** Summary of the results obtained for cardiac muscle

**No. of Chains in parallel**	**TPT (msec)**			**Transverse velocity (cm/sec)**			**Overall velocity (cm/sec)**			**Ratios**		
	
	**0/0**	**100/0**	**100/100**	**0/0**	**100/0**	**100/100**	**0/0**	**100/0**	**100/100**	**A/C**	**F/D**	**I/G**
	**A**	**B**	**C**	**D**	**E**	**F**	**G**	**H**	**I**			
**3**	15.6	6.6	1.9	0.21	0.48	1.68	27.9	65.9	229	8.2	8.0	8.2
**5**	17.1	11.0	3.9	0.37	0.58	1.64	43.0	66.8	189	4.4	4.4	4.4
**7**	18.4	12.9	5.8	0.52	0.74	1.66	56.3	80.2	178	3.2	3.2	3.2
**10**	17.6	16.8	8.6	0.82	0.86	1.67	84.4	88.4	173	2.0	2.0	2.0
**20**	18.0	17.2	15.2	1.69	1.77	2.00	166	174	196	1.2	1.2	1.2

Each chain consisted of 10 cells, and the number of chains placed in parallel was varied from 3 to 20.

The number of gj-channels, longitudinally and transversely, is indicated by the column headings: 0/0, 100/0, and 100/100.

TPT is the total propagation time, measured as the elapsed time between when the first AP and last AP crossed the zero potential level.

Overall velocity (both longitudinal and transverse components) was calculated from the measured TPT and the distance traveled. Each myocardial cell was assumed to be 150 μm in length and 16 μm in diameter.

## References

[B1] Sperelakis N, Mann JE (1977). Evaluation of electric fieldchanges in the cleft between excitable cells. J Theor Biol.

[B2] Picone JB, Sperelakis N, Mann JE (1991). Expanded model of the electric field: Hypothesis for propagation in cardiac muscle. Math Computer Modeling.

[B3] Sperelakis N, Ramasamy L (2002). Propagation in cardiac muscle and smooth muscle based on electric field transmission at cell junctions: An analysis by PSpice. IEEE-Eng Med Biol.

[B4] Sperelakis N (2002). An electric field mechanism for transmission of excitation between myocardial cells. Circ Res.

[B5] Hogues H, Leon LJ, Roberge FA (1992). A model study of electric field interactions between cardiac myocytes. IEEE Trans Biomed Eng.

[B6] Rohr S (2004). Role of gap junctions in the propagation of the cardiac action potential. Cardiovasc Res.

[B7] Rohr S, Kucera JP, Fast VG, Kleber AG (1997). Paradoxical improvement of impulse conduction in cardiac tissue by partial cellular uncoupling. Science.

[B8] Sperelakis N (2001). Cell Physiology Sourcebook. 3^rd^edition. Chapter 24: Cable properties and propagation of action potentials. Appendix I: Academic Press.

[B9] Cohen SA (1994). Immunocytochemical localization of rH1 sodium channel in adult rat heart atria and ventricle. Presence in terminal intercalated disks. Circulation.

[B10] Kucera JP, Rohr S, Rudy Y (2002). Localization of sodiumchannels in intercalated disks modulates cardiac conduction. Circ Res.

[B11] Sperelakis N, Murali KPV (2003). Combined electric field and gap junctions on propagation of action potentials in cardiac muscle and smooth muscle in PSpice simulation. J Electrocardiol.

[B12] Morley GE, Vaidya D, Samie FH, Lo CW, Taffet SM, Delmar M, Jalife J (1999). Characterization of conduction in the ventricles of normal and heterozygous Cx43 knockout mice using optical mapping. J Cardiovasc Electrophysiol.

[B13] Tamaddon HS, Vaidya D, Simon AM, Paul DL, Jalife J, Morley GE (2000). High resolution optical mapping of the right bundle branch in connexin40 knockout mice reveals low conduction in the specialized conduction system. Circ Res.

[B14] Gutstein DE, Morley GE, Tamaddon H, Vaidya D, Schneider MD, Chen J, Chien KR, Stuhlmann H, Fishman GI (2001). Conduction slowing and sudden arrhythmic death in mice with cardiac restricted inactivation of connexin43. Circ Res.

[B15] Vaidya D, Tamaddon HS, Lo CW, Taffet SM, Delmar M, Morley GE (2001). Null mutation of connexin43 causes slow propagation of ventricular activation in the late stages of mouse embryonic development. Circ Res.

[B16] Sperelakis N, Kalloor B, Ramasamy L (2005). Boundary effects influence velocity of transverse propagation of simulated cardiac action potentials. Theor Biol & Med Modeling.

[B17] Sperelakis N (2003). Propagation of action potentials between parallel chains of cardiac muscle cells in PSpice simulation. Can J Physiol Pharmacol.

[B18] Sperelakis N, Kalloor B (2004). Transverse propagation of action potentials between parallel chains of cardiac muscle and smoothmuscle cells in PSpice simulations. Biomed Eng Online.

[B19] Ramasamy L, Sperelakis N (2005). Gap-junction channels inhibit transverse propagation in cardiac muscle. Biomed Eng Online.

[B20] Wang S, Leon LJ, Roberge FA (1996). Interactions between adjacent fibers in a cardiac muscle bundle. Ann Biomed Eng.

[B21] Sperelakis N, Ramasamy L (2002). Propagation in cardiac muscle and smooth muscle based on electric field transmission at cell junctions: An analysis by PSpice. IEEE-Eng Med Biol.

[B22] Ramasamy L, Sperelakis N (2005). Repolarization of the action potential enabled by Na+ channel deactivation in PSpice simulation of cardiac muscle propagation. Theor Biol & Med Modeling.

[B23] Koura T, Hara M, Takeuchi S, Ota K, Okada Y, Miyoshi S, Watanabe A, Shiraiwa K, Mitamura H, Kodama I, Ogawa S (2002). Anisotropic conduction properties in canine atria analyzed by high-resolution opticalmapping. Circulation.

[B24] Ramasamy L, Sperelakis N (2006). Effect of transverse gap-junction channels on transverse propagation in an enlarged PSpice model of cardiac muscle. Theor Biol Med Modeling.

[B25] Sperelakis N, Kalloor B (2004). Effect of variation in membrane excitability on propagation velocity of simulated action potentials for cardiac muscle and smooth muscle in the electric field model for cell to cell transmission of excitation. IEEE-Eng Med Biol.

[B26] Poelzing S, Akar FG, Baron E, Rosenbaum DS (2003). Heterogeneous connexin43 expression produces electrophysiological heterogeneities across ventricular wall. Am J Physiol: Heart & Circ Physiol.

[B27] Poelzing S, Rosenbaum DS (2004). Altered connexin43 expression produces arrhythmia substrate in heart failure. Am J Physiol: Heart & Circ Physiol.

[B28] Diaz PJ, Rudy Y, Plonsey R (1983). Intercalated discs as a cause for discontinuous propagation in cardiac muscle: A theoretical simulation. Ann Biomed Eng.

[B29] deCastro M, Hofer E, Munuzuri AP, Gomez-Gesteira M, Plank G, Schafferhofer I, Perez-Munuzuri V, Perez-Villar V (1999). Comparison between the role of discontinuities in cardiac conduction and in a one-dimensional hardware model. Physical Review E.

[B30] Henriquez AP, Vogel R, Muller-Borer BJ, Henriquez CS, Weingart R, Cascio WE (2001). Influence of dynamic gap junction resistance on impulse propagation in ventricular myocardium: a computer simulation study. Biophys J.

[B31] Spach MS, Heidlage JF The stochastic nature of cardiac propagation at a microscopic level: electrical description of myocardial architecture and its application to conduction. Circ Res.

